# A presentation of brown tumor in a patient with parathyroid adenoma and end-stage renal disease: A case report

**DOI:** 10.1097/MD.0000000000044342

**Published:** 2025-09-05

**Authors:** Ali Gohar, Muhammad Husnain Ahmad, Momina Masroor, Masab Ali, Ayesha Ayman, Ali Usman, Zainab Riaz, Bilal Ahmed

**Affiliations:** a Department of Internal Medicine, Lahore General Hospital, Lahore, Punjab, Pakistan; b Department of Medicine, Tentishev Satkynbai Memorial Asian Medical Institute, Kant, Kyrgyzstan; c Department of Kidney Transplant, Pakistan Kidney and Liver Institute and RC, Pakistan; d Department of Medicine, Punjab Medical College, Faisalabad, Pakistan.

**Keywords:** brown tumor, end-stage renal disease (ESRD), hyperparathyroidism, lytic bone lesions, maintenance hemodialysis (MHD), osteitis fibrosa cystica, parathyroid adenoma, technetium-99m sestamibi scan

## Abstract

**Rationale:**

Brown tumor (osteitis fibrosa cystica) is a benign bone lesion associated with hyperparathyroidism that can affect multiple bones in patients with end-stage renal disease (ESRD).

**Patient concerns:**

We present the case of a 32-year-old female with ESRD on maintenance hemodialysis who experienced body aches, muscle weakness, constipation, and mood swings for 3 months.

**Diagnoses:**

Initial tests revealed elevated parathyroid hormone (PTH), serum calcium, and phosphorus levels. A technetium-99m sestamibi scan identified a parathyroid adenoma in the inferior pole of the left thyroid lobe.

**Interventions:**

Despite conservative management, she underwent parathyroidectomy 2 years later, with biopsy showing parathyroid hyperplasia. Postoperatively, her symptoms persisted, and labs indicated elevated PTH, low serum calcium, and normal phosphorus. A repeat sestamibi scan detected a new adenoma in the right thyroid lobe and a brown tumor in the mandible. X-ray showed a left humerus fracture, and a bone scan revealed increased uptake in the skull, mandible, and multiple joints.

**Outcomes:**

The presence of a parathyroid adenoma, elevated PTH levels, chronic kidney disease requiring dialysis, and specific imaging findings suggest a potential diagnosis of brown tumor of the bone. This case illustrates the complexity of managing hyperparathyroidism in ESRD, particularly in younger patients. The clinical presentation can vary, with symptoms mimicking metastatic disease. Even after parathyroidectomy, the condition may not fully resolve.

**Lessons:**

Early recognition and intervention of brown tumors in ESRD patients on maintenance hemodialysis is crucial, especially when they present with elevated calcium and phosphorus levels.

## 1. Introduction

Brown tumors are rare, nonneoplastic lesions that result from abnormal bone metabolism due to hyperparathyroidism.^[1,2]^ They are characterized by osteoclastic resorption of bone, leading to fibroblastic proliferation, hemorrhage, and hemosiderin deposition, which gives the lesion a characteristic brown color.^[3]^ Brown tumors are observed in approximately 2% of individuals with primary hyperparathyroidism.^[1]^ The mandible is the most common site, representing 4% of cases with brown tumors, although the maxilla can also be affected.^[1]^ These lesions are often associated with primary hyperparathyroidism and secondary hyperparathyroidism. Primary hyperparathyroidism is commonly caused by parathyroid adenoma which leads to production of excessive PTH^[4]^ whereas, secondary hyperparathyroidism is a compensatory mechanism in chronic calcium-wasting conditions like chronic kidney disease (CKD).^[3]^ In rare instances, these novel tumors occur in presence of tertiary hyperparathyroidism as described by Topal. et all.^[5]^ The presentation of brown tumors can be varied and sometimes misleading, complicating early diagnosis and appropriate management. The initial investigations such as parathormone (PTH), alkaline phosphatase (ALP), serum calcium and serum phosphate can point to the metabolic bone disease while 99mTechnetium- methylenediphosphonate (MDP) bone scan and 99m Technetium- sestamibi whole body scan are useful in differentiating and managing these conditions.^[2,4]^ This case report aims to focus attention on the importance of considering brown tumors in the differential diagnosis of patients with ESRD and hyperparathyroidism, while also highlighting the diagnostic challenges and treatment outcomes associated with these lesions. By sharing this case, we hope to contribute valuable insights that can aid clinicians in recognizing and managing similar cases in the future.

## 2. Case description

A 32-year-old Asian female presented to the outpatient department of a Tertiary Care Hospital with complaints of abdominal pain, generalized body aches, muscle weakness, constipation, and frequent mood swings for 3 months. She had a prior history of hypertension leading to CKD with end-stage renal disease (ESRD), for which she had been on maintenance hemodialysis for 8 years.

A provisional diagnosis of hyperparathyroidism secondary to CKD was made. Initial biochemical laboratory tests revealed elevated PTH (parathyroid hormone), hypercalcemia, hyperphosphatemia and elevated creatinine. The values were as follows: calcium 9.1 mg/dL (normal range: 8.5–10.5 mg/dL), phosphate 7.4 mg/dL (normal range: 2.5–4.5 mg/dL), PTH 2190 pg/mL (normal range: 10–65 pg/mL) and creatinine 9.6 mg/dL (normal range: 0.6–1.3 mg/dL). In addition to hyperparathyroidism-related findings, the patient’s renal function was closely monitored throughout her treatment. Laboratory tests showed persistently elevated serum creatinine levels, hyperphosphatemia, anemia, increased PTH levels and Grade 2 parenchymal echogenicity on ultrasound kidney, ureter, bladder (KUB) indicative of CKD. The estimated glomerular filtration rate (eGFR) remained below 15 mL/min/1.73 m², consistent with ESRD. Dialysis adequacy was evaluated using standard parameters, with the patient’s Kt/V consistently above 1.2 and urea reduction ratio exceeding 65%, indicating effective hemodialysis sessions.

**Figure 1.**
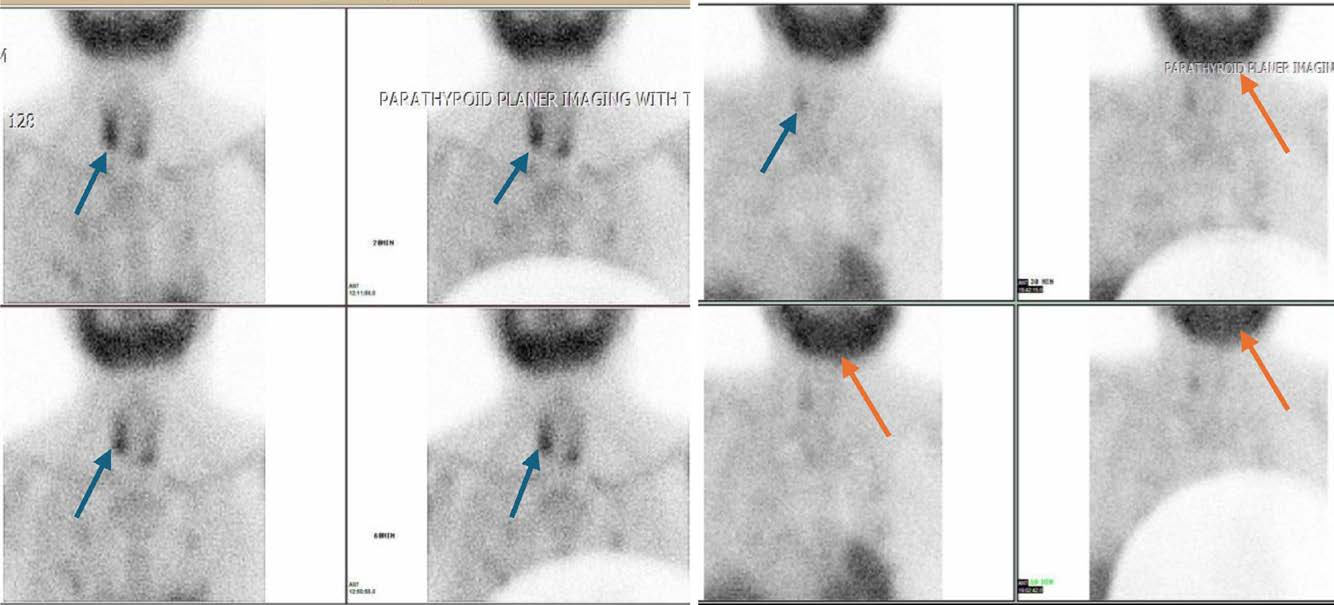
(A) Preoperative Tc99m MIBI parathyroid scan, revealing increased uptake in the parathyroid gland below the inferior pole of the left thyroid lobe, indicating a hyperfunctioning parathyroid gland (B) Postoperative Tc99m MIBI parathyroid scan showing brown tumor of mandible pointed by orange arrows. MIBI = methoxyisobutylisonitrile.

An ultrasound of the neck showed an oval-shaped hypoechoic nodule on the posterior aspect of the right thyroid lobe and a well-defined rounded cystic nodule adjacent to the lower pole of the left thyroid lobe, suggestive of an adenoma or lymph node. CT scan findings were consistent with a hyperfunctioning parathyroid gland inferior to the lower pole of the left thyroid lobe. The right intrathyroidal lesion was suspected to be a hyperfunctioning intrathyroidal parathyroid gland versus a thyroid follicular lesion. Subsequently, a 99mTc methoxyisobutylisonitrile parathyroid scan was performed, revealing increased uptake in the parathyroid gland below the inferior pole of the left thyroid lobe, indicating a hyperfunctioning parathyroid gland (Fig. 1A).

The patient was advised to undergo a parathyroidectomy but was reluctant to undergo surgery. She was already taking cinacalcet 30 mg BD, and her hyperparathyroidism (HPT) was managed medically. Her dialysis sessions continued, and her laboratory parameters were periodically monitored, showing persistently high PTH levels. The data from the onset of HPT detection to date are presented in Table 1.

**Table 1:** Laboratory data from onset of HPT detection until date.

Dates	Calcium (mg/dL)	Phosphate (mg/dL)	PTH (pg/mL)
August 2021	9.1	7.4	2412
July 2022	7.53	2.7	2543
July 2023	9.78	4.7	2324
March 2024	9.31	6.3	2898

HPT = hyperparathyroidism, PTH = parathyroid hormone.

**Figure 2.**
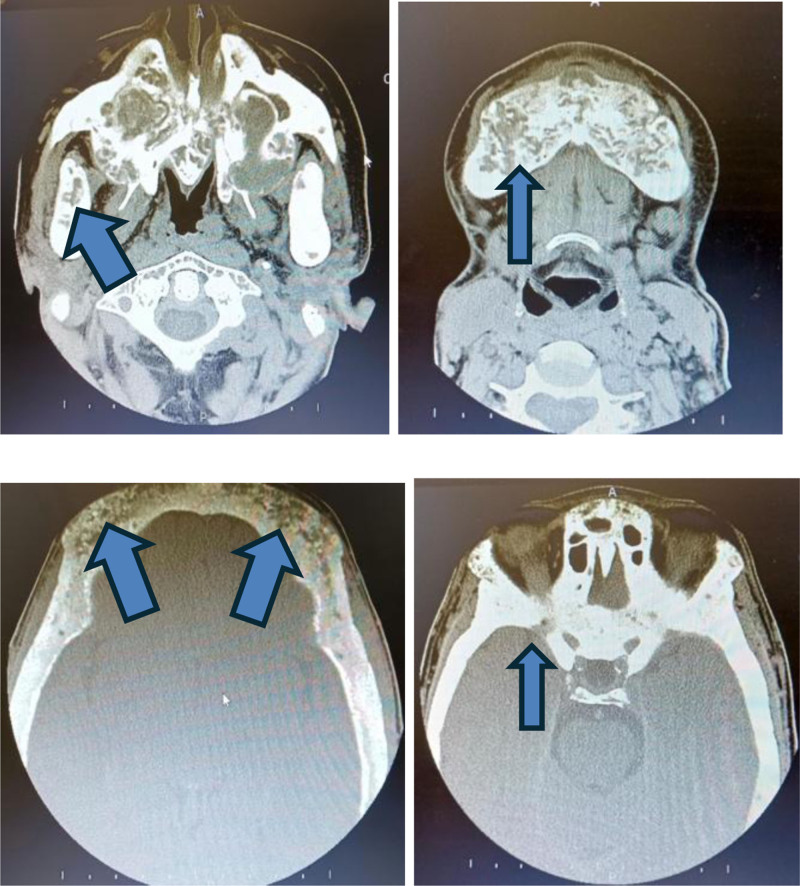
CT paranasal sinuses – FESS protocol showing lytic lesions of skull and facial Bones pointed by blue arrows. CT = computed tomography, FESS = functional endoscopic sinus surgery.

Her generalized weakness progressively worsened over the period of 2 years. On examination, she had kyphoscoliosis. A CT scan of the paranasal sinuses using the functional endoscopic sinus surgery protocol showed a well-defined soft tissue mass inferior to the lower pole of the left thyroid lobe, measuring 1.1 × 1.2 cm, and a relatively hypodense nodule within the lower half of the right thyroid lobe, measuring approximately 1.8 × 1.0 cm. Bilateral lungs, mediastinum, and upper abdominal structures appeared grossly unremarkable. Multiple reactive-looking nodes were seen in the bilateral axillae. Osteopenic changes were noted in the visualized skeleton (Fig. 2).

Her parathyroidectomy was performed, during which 2 parathyroid glands were removed: one from the inferior pole of the left thyroid lobe and another from the upper pole of the right thyroid lobe. Preoperative PTH levels were recorded at 2898 pg/mL. Postoperative biopsy confirmed hyperplasia of the parathyroid glands. Postoperatively, her laboratory results revealed persistently elevated PTH levels, persistently low serum calcium, and normal phosphorus. Her lab values were: calcium 4.49 mg/dL (normal range: 8.5–10.5 mg/dL), phosphorus 3.8 mg/dL (normal range: 2.5–4.5 mg/dL), and PTH 1089 pg/mL (normal range: 10–65 pg/mL).

**Figure 3.**
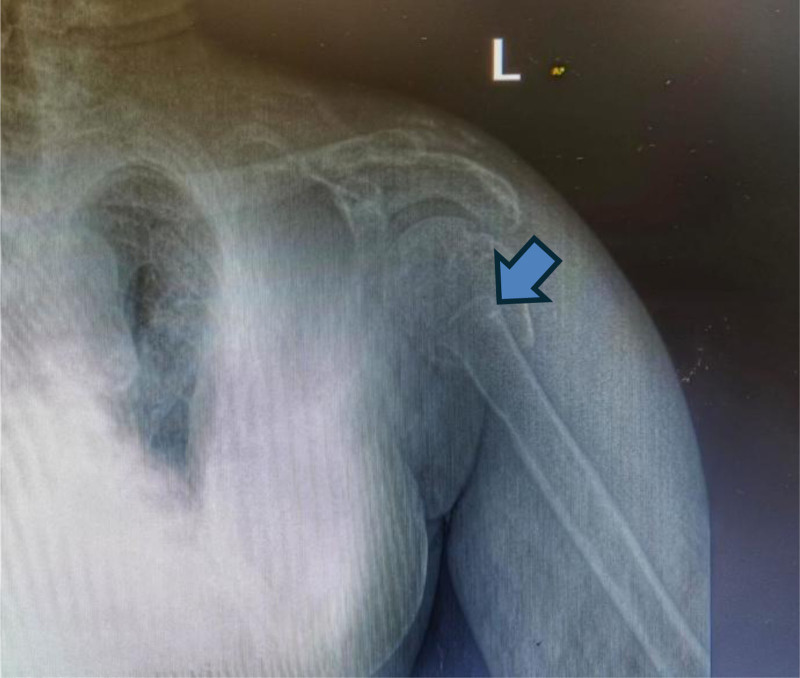
X-ray left shoulder joint showing left humerus fracture pointed by blue arrow.

**Figure 4.**
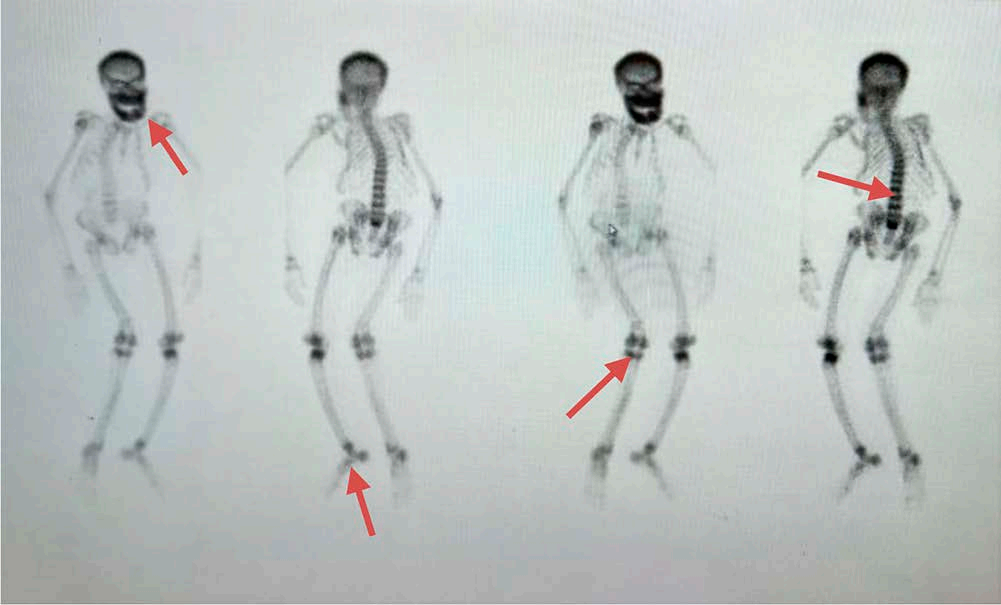
Bone scan showing increased uptake in skull, mandible (Abraham Lincoln Sign) and multiple joints as pointed by orange arrows.

99mTc methoxyisobutylisonitrile parathyroid scan was repeated and revealed evidence of intensely increased radiotracer uptake in the upper pole of the right lobe of the thyroid and the whole of the mandible bone, suggesting a possible osteoblastic tumor of bone (Fig. 1B). A completely displaced overriding fracture of the proximal shaft of the left humerus is noted on the x-ray of the left shoulder joint. There is heterogeneously increased bone density, which may suggest underlying renal osteodystrophy changes (Fig. 3). Bone scan 99mTc MDP revealed intensely increased radiotracer uptake in the skull, mandible (Abraham Lincoln sign), and multiple joints, most likely due to renal osteodystrophy (Fig. 4).

The findings of parathyroid adenoma, high levels of PTH, CKD on dialysis, and imaging results raised the possibility of a brown tumor of the bone. The patient has been non-compliant with follow-up due to personal reasons best known to her.

## 3. Discussion

A rare subtype of nonneoplastic lesions that result from excessive osteoclastic activity, abnormal fibroblastic proliferation, the admixture of osteoclasts reactive giant cells and hemorrhagic debris forming a specific hemosiderin-laden brown mass is called brown tumor. It is a well-documented complication of long-standing, uncorrected hyperparathyroidism, typically seen in older individuals.^[1,3,6]^ A few differentials are usually considered with such presentation such as osteoclastoma and metastatic tumors.^[7,8]^ However, our case, involving a 32-year-old female with ESRD, more inclines towards the diagnosis of brown tumor which was later confirmed with appropriate investigations, highlights its occurrence in a younger population. The patient’s secondary hyperparathyroidism due to CKD progressed to tertiary hyperparathyroidism, evident from increased parathyroid activity in the left lower and right upper thyroid lobes.

Abnormal calcium levels significantly impact renal function in ESRD patients. Hypercalcemia promotes vascular calcifications, particularly in coronary arteries, increasing cardiovascular risks and impairing dialysis efficiency.^[3]^ Conversely, hypocalcemia, often observed post-parathyroidectomy or during hyperparathyroidism management, exacerbates secondary hyperparathyroidism, worsening bone resorption and renal osteodystrophy. Both conditions contribute to systemic complications, emphasizing the importance of early detection and management to improve outcomes in ESRD patients.^[1,6]^

Singh et al reported a CKD stage 4 patient presenting with painful jaw swelling, whereas our patient had no jaw swelling, only hypercalcemia-related symptoms. This underscores the variability in brown tumor presentation, necessitating high clinical suspicion.^[9]^ Wadhawan et al described cases with normal calcium levels presenting as facial swelling, contrasting with our patient’s hypercalcemia and nonspecific findings, further complicating diagnosis.^[1]^ Physicians must consider both typical and atypical presentations to avoid diagnostic delays.

A case by Tbini et al emphasized the diagnostic challenges in brown tumors presenting as palatal swellings in patients with secondary hyperparathyroidism. Imaging and biochemical markers were critical in differentiating these lesions from other conditions, a practice that was also key in our case.^[10]^ Similarly, Belkouchi et al highlighted that massive brown tumors of the jaw could mimic malignancies, emphasizing the importance of comprehensive imaging and histopathological evaluation, aligning with the approach taken in our patient’s management.^[11]^

Treatment for brown tumors primarily involves medical therapy to normalize PTH, calcium, and phosphate levels. Although cinacalcet was prescribed, our patient’s persistently elevated PTH levels suggest that medical management alone may be insufficient.^[3]^ As noted by Srikantha et al, surgical intervention is often required. Our patient underwent parathyroidectomy, during which 2 glands were removed. However, postoperatively, PTH levels remained elevated, and calcium levels were persistently low, indicating that even surgery may not always yield expected outcomes^[1,3]^

The interplay between abnormal calcium-phosphorus metabolism and renal function in ESRD is critical. Hypercalcemia and secondary hyperparathyroidism can lead to vascular calcifications, further impairing renal perfusion and contributing to a decline in kidney function.^[3]^ In our patient, despite regular hemodialysis and medical management, persistently elevated parathyroid hormone (PTH) levels exacerbated bone turnover and calcium-phosphorus imbalance, complicating the overall management. Monitoring dialysis adequacy (e.g., Kt/V and urea reduction ratio) alongside biochemical markers is essential for optimizing outcomes in such patients.^[1]^

Diagnostic imaging plays a pivotal role. Tc-99m sestamibi scans identified hyperfunctioning parathyroid glands in our case, consistent with findings from Wadhawan et al Functional cysts like parathyroid adenomas are less likely to be detected on scintigraphy, highlighting the importance of combining imaging with biochemical markers.^[9]^ Additionally, multiple lytic bone lesions may mimic metastatic disease, complicating diagnosis, as noted in studies by Fedhila et al.^[12]^

Early detection and prevention require routine monitoring of biochemical markers (PTH, calcium, phosphate) and imaging studies. Calcimimetics and phosphate binders help manage imbalances, while timely parathyroidectomy prevents progression. Multidisciplinary care involving nephrologists, endocrinologists, and surgeons ensures comprehensive management, improving patient outcomes.^[3,9]^

Wadhawan et al reported in their case study that the PTH levels of the patient plummeted after undergoing parathyroidectomy and the brown tumor also reduced by a considerable amount which strikingly contrasts with our case in which the labs showed persistently raised PTH and low calcium and normal phosphorus highlighting the fact that even parathyroidectomy may not be adequate sometimes for the treatment of hyperparathyroidism.^[1]^

## 4. Conclusion

Brown tumors of the mandible can occur in patients with CKD due to secondary or tertiary hyperparathyroidism. These tumors may mimic other conditions, making accurate diagnosis and timely intervention critical. It highlights the potential limitations of standard treatments and the importance of documenting such cases to enhance clinical understanding and management strategies. A multidisciplinary approach involving endocrinologists, nephrologists, and nuclear physicians is essential for optimal patient care. Collaboration among these specialists ensures comprehensive management, including effective treatment of hyperparathyroidism, appropriate surgical interventions, and ongoing monitoring of bone health. This integrated strategy improves patient outcomes by addressing the complex interplay of endocrine, renal, and skeletal issues.

## Acknowledgments

We would like to thank the team of clinicians who helped manage this case. We would like to thank the patient and his family members for their cooperation in bringing this case for the betterment of the scientific community.

## Author contributions

**Conceptualization:** Ali Gohar, Zainab Riaz.

**Data curation:** Ali Gohar.

**Investigation:** Zainab Riaz.

**Software:** Ayesha Ayman.

**Validation:** Momina Masroor, Ayesha Ayman, Masab Ali.

**Visualization:** Momina Masroor, Masab Ali, Muhammad Husnain Ahmad, Ali Usman, Bilal Ahmed.

**Writing – original draft:** Masab Ali, Muhammad Husnain Ahmad, Ali Usman.

**Writing – review & editing:** Masab Ali, Bilal Ahmed.
